# Severe Hepatitis in a Patient With Undifferentiated Connective Tissue Disease: Diagnostic Uncertainty Between Methotrexate Toxicity and Autoimmune Liver Involvement

**DOI:** 10.7759/cureus.97526

**Published:** 2025-11-22

**Authors:** Bola Reyad

**Affiliations:** 1 General Internal Medicine, St. Mary's Hospital, Isle of Wight NHS Trust, Isle of Wight, GBR

**Keywords:** autoimmune hepatitis, autoimmune liver disease, connective tissue disease, drug induced liver injury, hepatotoxicity, jaundice, lupus hepatitis, methotrexate toxicity, undifferentiated connective tissue disease

## Abstract

A patient in their mid-20s with a background of undifferentiated connective tissue disease (UCTD) presented with two days of worsening jaundice, pruritus, and vomiting. Methotrexate had been stopped six weeks earlier because of mildly elevated liver enzymes, yet the abnormalities progressed after withdrawal. On examination, there was marked jaundice and a non-specific maculopapular rash involving the limbs and face. Laboratory studies showed alanine aminotransferase (ALT) 1,500 IU/L, aspartate aminotransferase (AST) 1,200 IU/L, and bilirubin 238 µmol/L, with normal international normalised ratio (INR) and renal function. Viral, metabolic, and toxicology screens were negative. Autoimmune tests revealed weakly positive antinuclear antibody (ANA) but no other antibodies, with normal immunoglobulin G (IgG )and complement levels. Imaging excluded obstruction. The rash resolved and liver tests normalised within one week with supportive management alone.

This case highlights the diagnostic overlap between methotrexate-induced hepatotoxicity and autoimmune-related liver injury in patients with connective tissue disease. Methotrexate toxicity can sometimes persist or worsen after discontinuation, whereas lupus-related hepatitis often lacks strong serological markers. In this patient, the absence of cytopenias, mucosal ulceration, or raised IgG argued against classic methotrexate toxicity or autoimmune hepatitis. Spontaneous recovery without corticosteroids suggested a transient, self-limiting autoimmune process. Careful clinical correlation, multidisciplinary review, and cautious monitoring can help avoid unnecessary escalation of immunosuppressive therapy in similar presentations.

## Introduction

Liver dysfunction in patients with autoimmune connective tissue diseases presents a well-recognised diagnostic challenge because several mechanisms can coexist, including drug-induced hepatotoxicity, classical autoimmune hepatitis, and hepatic inflammation secondary to systemic autoimmune activity [1-3]. Determining the underlying cause is clinically important, as management decisions and long-term outcomes differ markedly among these conditions.

Methotrexate remains a cornerstone therapy in many autoimmune and inflammatory disorders, including undifferentiated connective tissue disease (UCTD). Liver enzyme abnormalities during therapy are well-described and generally improve after dose adjustment or withdrawal, although a subset of patients may continue to deteriorate biochemically after cessation. In such scenarios, an overlapping or reactive immune-mediated process rather than direct hepatotoxicity should be considered [4-6].

Lupus-related hepatitis, also referred to as lupus hepatopathy, is relatively uncommon but often under-recognised. It occurs most frequently in systemic lupus erythematosus (SLE) and may also appear in incomplete or undifferentiated autoimmune syndromes [7,8]. The clinical and biochemical features often mimic both autoimmune hepatitis and drug-induced liver injury, complicating diagnostic evaluation. Because treatment decisions, particularly whether to initiate corticosteroids or other immunosuppressants, depend on distinguishing these mechanisms, multidisciplinary assessment is pivotal.

This report describes a young patient with UCTD who developed acute hepatitis shortly after discontinuing methotrexate. The case illustrates the overlap between methotrexate-related hepatotoxicity and immune-mediated hepatic injury and highlights how careful reasoning and coordinated multidisciplinary care can guide safe, conservative management without unnecessary immunosuppression.

## Case presentation

A patient in their mid-20s presented with 2 days of progressive jaundice, generalised pruritus, and persistent vomiting. Past medical history included UCTD and chronic idiopathic urticaria. UCTD had been diagnosed two years earlier following recurrent arthralgia, intermittent rashes, and a positive antinuclear antibody (ANA) titre of 1:160, with a speckled pattern. Complement levels were normal, and extractable nuclear antigen antibodies were negative. UCTD is recognised as an autoimmune overlap condition in which patients display features of connective tissue disease without fulfilling the classification criteria for SLE or another defined autoimmune syndrome [1,2]. The patient had remained clinically stable on low-dose methotrexate and folic acid for inflammatory joint pain.

Methotrexate (15 mg weekly) had been discontinued 6 weeks before admission after a routine test showed a mildly elevated alanine aminotransferase (ALT) of 120 IU/L. There was no history of alcohol use, recreational drugs, or herbal supplement intake, and no family history of liver disease. On examination, the patient was afebrile, alert, and haemodynamically stable. There was marked jaundice with mild scleral icterus. A faint, non-specific maculopapular rash was noted on the limbs and face. The abdomen was soft, with a palpable but non-tender liver edge. No splenomegaly, lymphadenopathy, or ascites was detected.

Initial investigations demonstrated markedly raised aminotransferases (ALT 1,500 IU/L; aspartate aminotransferase (AST) 1,200 IU/L), an alkaline phosphatase (ALP) of 225 IU/L, and total bilirubin of 238 µmol/L, with a normal international normalised ratio (INR) of 1.0. Renal function was preserved. Viral hepatitis serology (hepatitis A immunoglobulin M (IgM), hepatitis B surface antigen, anti-hepatitis C antibody, and hepatitis E IgM/IgG) and viral screens for Epstein-Barr virus, cytomegalovirus, and human immunodeficiency virus were all negative [3]. Autoimmune screening revealed weak ANA positivity (1:80) but negative anti-smooth-muscle and anti-liver/kidney microsomal type 1 (LKM1) antibodies. Serum immunoglobulins and complement levels were normal. Ferritin and caeruloplasmin were within normal limits, while iron studies showed a mildly elevated transferrin saturation (53%). Toxicology screening for paracetamol and salicylate was negative.

**Figure 1:**
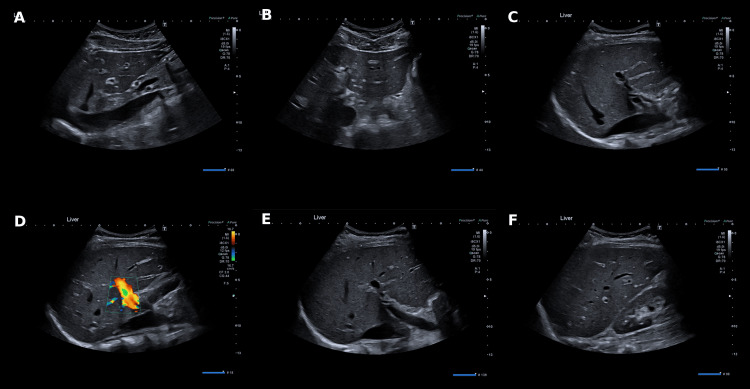
Abdominal ultrasound demonstrating normal hepatic architecture and biliary anatomy Transverse and longitudinal ultrasound images (A–F) of the upper abdomen. (A–C) Homogeneous hepatic echotexture with smooth contour and no focal lesions or intrahepatic duct dilatation. (D) Colour Doppler showing preserved portal venous flow with normal direction and velocity. (E–F) Gallbladder and common bile duct of normal calibre, with no wall thickening or gallstones. Overall appearances are normal, excluding biliary obstruction or structural liver disease as a cause of the patient’s jaundice.

A summary of the key laboratory findings, including both peak and discharge values, is presented in Table 1. Abdominal ultrasound (Figure 1) demonstrated normal hepatic architecture with homogeneous echotexture, no biliary dilatation, and no focal lesions. The gallbladder, spleen, pancreas, and both kidneys appeared normal, with no evidence of hepatic congestion or portal hypertension. These findings supported a non-obstructive hepatocellular process.

**Table 1:** Summary of laboratory findings during acute presentation and at discharge Laboratory findings showed a clear hepatocellular injury pattern with marked transaminase elevation, normal coagulation profile, and preserved renal and synthetic function. Rapid biochemical improvement was observed by discharge, consistent with a self-limiting immune-mediated hepatic process.

Parameter	Peak Value	Discharge Value	Units	Reference Range
Haemoglobin	150	148	g/L	115–160
Mean Cell Volume	88	88	fL	80–100
White Cell Count	6.6	6.4	×10⁹/L	4.0–11.0
Platelets	286	290	×10⁹/L	150–400
Sodium	141	140	mmol/L	133–146
Potassium	3.7	4	mmol/L	3.5–5.1
Urea	4.3	4.1	mmol/L	2.5–7.8
Creatinine	58	60	µmol/L	45–90
Adjusted Calcium	2.4	2.38	mmol/L	2.20–2.60
Phosphate	1.24	1.2	mmol/L	0.8–1.5
Total Bilirubin	238	68	µmol/L	<21
Alanine Aminotransferase (ALT)	1,487	210	IU/L	<45
Aspartate Aminotransferase (AST)	1,200	165	IU/L	<40
Alkaline Phosphatase (ALP)	135	120	IU/L	40–130
Albumin	32	35	g/L	35–50
C-Reactive Protein (CRP)	12	5	mg/L	<5
Estimated Glomerular Filtration Rate (eGFR)	>90	>90	mL/min/1.73 m²	>60
International Normalised Ratio (INR)	1	1	—	0.8–1.2

The dermatology team reviewed the rash and deemed it non-specific. A biopsy was discussed but deferred, as the lesions resolved spontaneously within a few days.

The patient was managed conservatively with intravenous fluids, antiemetics, and cholestyramine for pruritus. Corticosteroids and other immunosuppressive agents were withheld. Over the next five days, ALT decreased from approximately 1,500 IU/L to 210 IU/L, and bilirubin fell from 238 µmol/L to 68 µmol/L, indicating rapid biochemical recovery. The patient’s symptoms resolved completely, and they were discharged on day seven with outpatient follow-up arranged in both hepatology and rheumatology clinics.

## Discussion

Differential diagnosis

Methotrexate-Induced Hepatotoxicity

Methotrexate is widely used in autoimmune and inflammatory disorders, but its potential to cause hepatic injury is well-recognised. The degree of hepatotoxicity may range from mild, transient transaminase elevation to progressive fibrosis with prolonged exposure. Hepatic dysfunction usually improves after discontinuation; however, in some cases, deterioration can continue due to residual metabolic injury or delayed drug clearance [5,6,8].

In this patient, there were no features typical of methotrexate toxicity such as pancytopenia, mucosal ulceration, or coagulopathy. Furthermore, the progressive worsening of liver enzymes several weeks after stopping the drug made direct drug-induced hepatotoxicity less likely.

Autoimmune or Lupus-Related Hepatitis

Autoimmune hepatitis typically presents with elevated aminotransferases, hypergammaglobulinaemia, and the presence of specific autoantibodies such as anti-smooth muscle or anti-liver/kidney microsomal type 1 (LKM1) [3,7]. None of these features was present in this case. However, the weak ANA positivity, history of undifferentiated connective tissue disease, and concurrent rash suggested an underlying immune-mediated mechanism. The spontaneous biochemical and clinical recovery without corticosteroid therapy supports a transient, self-limiting autoimmune flare rather than classical autoimmune hepatitis.

Clinical reasoning and management approach

UCTD is recognised as an autoimmune overlap condition in which patients display features of connective tissue disease but do not meet the established classification criteria for SLE [1,2]. Progression to defined lupus occurs only in a minority of cases. In this patient, SLE could not be confirmed, as the Systemic Lupus International Collaborating Clinics (SLICC) criteria were not met - there were no cytopenias, renal involvement, or disease-specific antibodies [9].

Given this diagnostic uncertainty, a multidisciplinary approach was adopted. The dermatology team assessed the rash, which was found to be non-specific and inconsistent with lupus or psoriasis. A skin biopsy was discussed but deferred once the lesions resolved spontaneously. Similarly, a liver biopsy was avoided because of clear biochemical improvement within a few days. Supportive management with intravenous fluids, antiemetics, and cholestyramine for pruritus proved sufficient, leading to full recovery without corticosteroids or further immunosuppressive therapy.

Lessons for clinical practice

This case underscores the importance of maintaining a broad differential diagnosis when assessing hepatic dysfunction in patients with autoimmune disease. When serological results are inconclusive but the clinical course is stable or improving, conservative management with close monitoring can prevent unnecessary invasive procedures and avoid overtreatment with corticosteroids or other immunosuppressants.

Effective communication and multidisciplinary collaboration between hepatology, rheumatology, and dermatology teams were key to ensuring safe, patient-centred care.

In summary, although lupus-related hepatitis could not be definitively confirmed, the temporal pattern of enzyme derangement, the autoimmune background, and the rapid spontaneous resolution all favour a transient, immune-mediated hepatic process rather than methotrexate toxicity. A cautious, coordinated approach allowed full recovery while avoiding unnecessary intervention.

## Conclusions

This case highlights the diagnostic uncertainty that can arise when hepatic dysfunction develops in a patient with autoimmune connective tissue disease. Differentiating between methotrexate-induced hepatotoxicity and immune-mediated liver injury required careful clinical reasoning, sequential investigations, and coordinated multidisciplinary input.

The patient’s spontaneous biochemical and clinical improvement following methotrexate withdrawal and supportive care suggested a transient immune-mediated hepatic reaction rather than classic methotrexate toxicity or autoimmune hepatitis. This report underscores the value of conservative management in clinically stable patients and demonstrates how effective collaboration between the hepatology, rheumatology, and dermatology teams can optimise diagnostic accuracy, avoid unnecessary interventions, and promote safe, patient-centred care.
